# Intracranial alternating current stimulation facilitates neurogenesis in a mouse model of Alzheimer’s disease

**DOI:** 10.1186/s13195-020-00656-9

**Published:** 2020-07-23

**Authors:** Qian Liu, Yihang Jiao, Weijian Yang, Beiyao Gao, Daniel K. Hsu, Jan Nolta, Michael Russell, Bruce Lyeth, Theodore P. Zanto, Min Zhao

**Affiliations:** 1 Department of Dermatology, Institute for Regenerative Cures, University of California at Davis, School of Medicine, Sacramento, CA 95817 USA; 2 Center for Neuroscience, Department of Neurological Surgery, School of Medicine, University of California at Davis, Sacramento, CA 95817 USA; 3 Department of Electrical and Computer Engineering, University of California at Davis, Davis, CA 95616 USA; 4 Present location: Department of Rehabilitation Medicine, Huashan Hospital, Fudan University, Shanghai, 200041 P. R. China; 5 Stem Cell Program and Gene Therapy Center, Institute for Regenerative Cures, Department of Internal Medicine, University of California at Davis, Sacramento, 95817 CA USA; 6 Neuroscape, Department of Neurology, University of California San Francisco – Mission Bay, Sandler Neuroscience Center MC 0444, San Francisco, CA 94158 USA; 7 Department of Ophthalmology and Vision Science, University of California at Davis, Sacramento, CA 95616 USA

**Keywords:** 5xFAD, Subventricular zone (SVZ), Hippocampus, Ki67, Nestin, Doublecortin (DCX), Intracranial electrical stimulation

## Abstract

**Background:**

Neurogenesis is significantly impaired in the brains of both human patients and experimental animal models of Alzheimer’s disease (AD). Although deep brain stimulation promotes neurogenesis, it is an invasive technique that may damage neural circuitry along the path of the electrode. To circumvent this problem, we assessed whether intracranial electrical stimulation to the brain affects neurogenesis in a mouse model of Alzheimer’s disease (5xFAD).

**Methods and results:**

We used Ki67, Nestin, and doublecortin (DCX) as markers and determined that neurogenesis in both the subventricular zone (SVZ) and hippocampus were significantly reduced in the brains of 4-month-old 5xFAD mice. Guided by a finite element method (FEM) computer simulation to approximately estimate current and electric field in the mouse brain, electrodes were positioned on the skull that were likely to deliver stimulation to the SVZ and hippocampus. After a 4-week program of 40-Hz intracranial alternating current stimulation (iACS), neurogenesis indicated by expression of Ki67, Nestin, and DCX in both the SVZ and hippocampus were significantly increased compared to 5xFAD mice who received sham stimulation. The magnitude of neurogenesis was close to the wild-type (WT) age-matched unmanipulated controls.

**Conclusion:**

Our results suggest that iACS is a promising, less invasive technique capable of effectively stimulating the SVZ and hippocampus regions in the mouse brain. Importantly, iACS can significantly boost neurogenesis in the brain and offers a potential treatment for AD.

## Background

Neurogenesis in both the subventricular zone (SVZ) and hippocampus in mammals has been linked to learning, memory, stress, and exercise [1–3] and is known to be impaired in neurological disease [4]. As such, stimulation of neurogenesis is a promising avenue to facilitate the remediation of disease and injured brains [5, 6]. In the adult human brain, neurogenesis drops significantly, even to undetectable levels in both the SVZ and hippocampus [5–12]. Yet, recent improvements in fixation and labelling techniques have demonstrated abundant hippocampal neurogenesis in the healthy adult human brain [4, 13]. Unfortunately, this neurogenesis is impaired in Alzheimer’s disease (AD), and as AD advances, the number and maturation of neurons decline progressively. Using doublecortin (DCX) as a biomarker of neurogenesis, it was recently shown that at Braak stages IV, V, and VI, DCX-positive cells in the hippocampus in AD brain were less than 25% of that in the hippocampus from healthy control brains [4]. Furthermore, maturation of DCX+ cells was found to be significantly impaired in the hippocampus from patients with AD. Therefore, defective neurogenesis in the AD brain may implicate memory and other functional deficits.

The finding of defective neurogenesis in the brains of AD patients is consistent with experimental studies using animal models of AD, in which significantly lower levels of neurogenesis are seen in both the SVZ and hippocampus [14]. Reduced neurogenesis has been reported in several AD animal models, including 5xFAD and Tg2576 mice, and OXYS rats [15–18].

Enhancing neurogenesis may improve cognition, and as such, many approaches have been assessed. Notably, physical exercises and non-steroidal anti-inflammatory drugs that upregulate neurotrophins have been reported to stimulate neurogenesis [19–24]. More recently, biophysical factors have been suggested to induce neurogenesis. Those include ultrasound, magnetic, and deep brain electrical stimulation [25–33]. Ultrasound stimulation is non-invasive and can induce hippocampal neurogenesis in healthy mice [29]. Deep brain electrical stimulation enhances metabolism, improves memory and behavior, and induces neurogenesis in the healthy and diseased rodent brain [34–37].

Unfortunately, all those available methods have various drawbacks preventing effective use in the clinic. For example, regular intense exercise is difficult to implement for patients (most often unable to do so independently), while stem cell therapy involves invasively injecting cells into the brain. Ultrasound compromises the blood-brain barrier permeability via injection of microbubbles [29], which carries the risk of tissue damage and behavioral decline [38]. Deep brain stimulation requires electrodes inside the brain, which induces injuries to the brain. Additionally, these treatments are very expensive.

Here, we aim to develop intracranial alternating current stimulation (iACS) as a mean to amplify neurogenesis in the AD brain. Although iACS has been used to safely enhance cognitive performance in both animals and humans [39–41], the effects of iACS on neurogenesis in AD models have not been described.

In this report, we assess the plausibility of using iACS to stimulate two sites of neurogenesis—the SVZ and the hippocampus, deep in the brain. A numerical model of iACS showed that stimulation can influence both the mouse SVZ and hippocampus. Next, we test the effects of iACS on neurogenesis in a mouse model of AD (5xFAD). We first determined that neurogenesis in 4-month-old 5xFAD mice was significantly decreased. We reasoned that this early stage of deficient neurogenesis would be responsive to show enhanced neurogenesis after iACS, if there are any effects to be observed. Therefore, we selected 3-month-old 5xFAD mice for iACS, in order to target this early stage of decline in the AD model brain. After a 4-week program of iACS (40 Hz), neurogenesis marked by Ki67, Nestin, and DCX in both the SVZ and hippocampus were significantly increased compared to 5xFAD mice who received sham stimulation. Furthermore, iACS facilitated neurogenesis to a level close to the WT age-matched control.

## Materials and methods

### Experimental animals and the Alzheimer’s disease mouse model

All experiments for this study were carried out following the procedures approved by the Institutional Animal Care and Use Committee at the University of California at Davis. The animals were housed in a temperature-controlled environment (22 ± 0.5 °C) with a 12-h-light-dark cycle and allowed free access to food and water. All efforts were made to minimize animal suffering and reduce the number of animals used.

The Alzheimer’s disease model mice were the 5xFAD transgenic mouse strain (B6.Cg-Tg (APPSwFlLon, PSEN1*M146L* L286V) 6799Vas/Mmjax), purchased from the Jackson Laboratory (RRID: MMRRC_034848-JAX). These mice carry the mutant human amyloid precursor protein (APP, 695) with the Swedish (K670N, M671L), Florida (I716V), and London (V717I) familial Alzheimer’s disease (FAD) mutations and human *PS1* harboring two FAD mutations, M146L and L286V. For the wild-type (WT) control model mice, we used age-matched C57BL/6J mice, because the 5xFAD strain is on a congenic C57BL/6J genetic background. Both 5xFAD and WT male mice at the age of 3 months were subjected to iACS to assess the effects on neurogenesis. The mice were divided into 3 groups: (1) WT sham treatment, (2) 5xFAD control, and (3) iACS-treated 5xFAD. For each group, 5 animals were used.

### Modeling iACS to target the hippocampus and SVZ

**Fig. 1:**
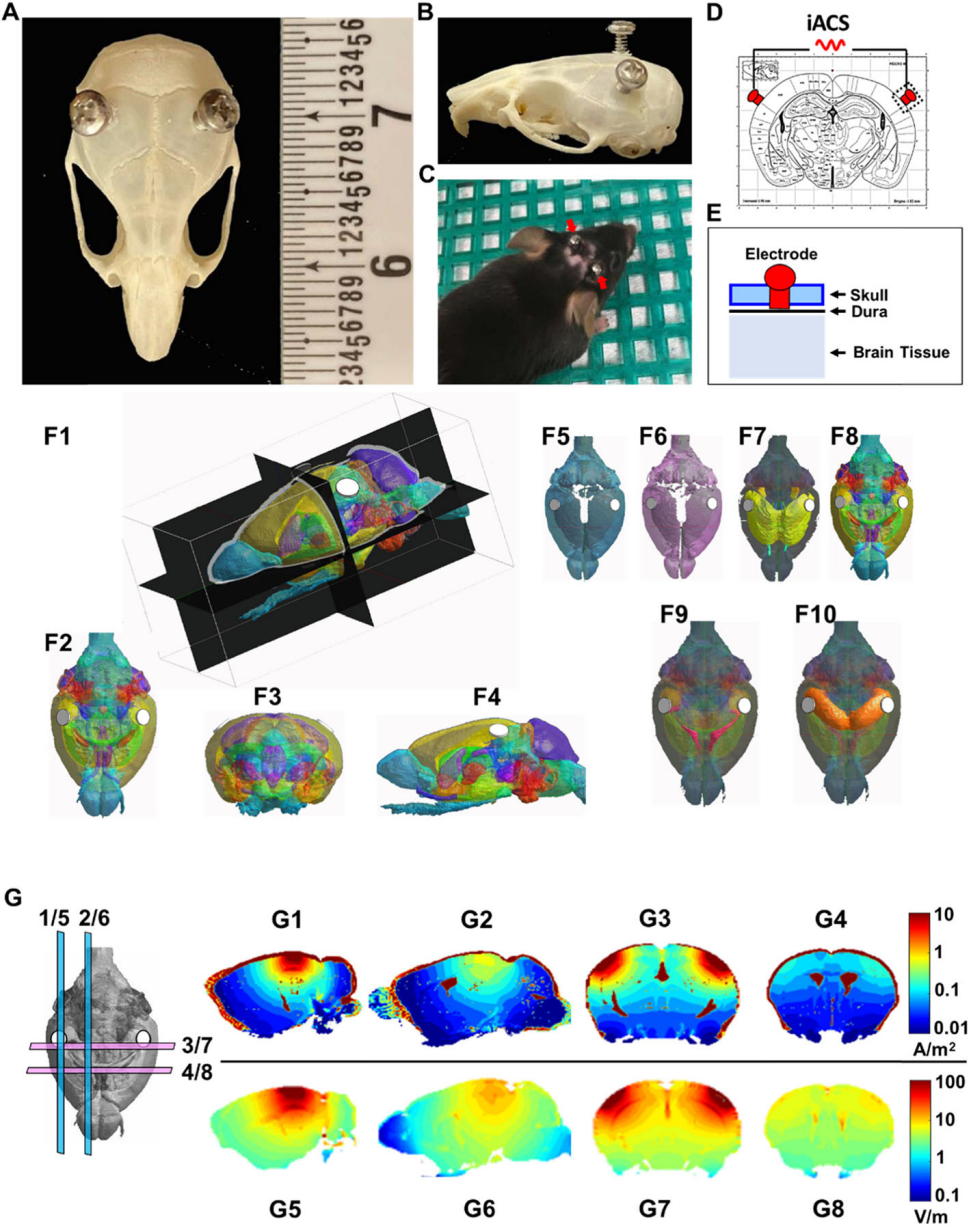
Intracranial AC stimulation and the estimated current distribution. **A**–**C** Two small stainless steel screws were implanted in the skull at anterior-posterior (AP) = − 2 mm and medial-lateral (ML) = 4 (left and right) mm to the bregma. **D**, **E** The iACS was delivered through the screw electrodes on the dura. The mouse brain atlas was quoted from ref. [42]. **F** The three-dimensional (3D) brain model, based on a C57BL/6 mouse brain atlas built from MRI and Nissl histology, which consists of 39 different brain segments (in different colors, **F1**). **F2**–**F4** The top (**F2**), front (**F3**), and side (**F4**) views of the 3D brain model with electrodes (white circles) on both hemispheres. **F5** The dura layer of the 3D brain model. **F6** The cerebral spinal fluid layer under the dura. **F7** The white matter of the 3D brain model in color (other brain regions were shown in gray shade). **F8** The gray matter of the 3D brain model in color (other brain regions were shown in gray shade). **F9** The lateral ventricle of the 3D brain model in pink (other brain regions were shown in gray shade). **F10** The hippocampus of the 3D brain model in orange (other brain regions were shown in gray shade). **G** Computer simulation was used to estimate the current densities (**G1**–**G4**, A/m^2^) and electric field strengths (**G5**–**G8**, V/m) in different brain regions, thus guide positioning of electrodes that would likely result in desirable and safe current and electric field distributions at sites of neurogenesis, including the subventricular zone (SVZ) and the hippocampus. The strongest currents and electric fields originate from the electrodes (circles in **G**) and flow into the brain with gradually decreasing density (**G1**, **G3**, **G5**, **G7**). The current of ~ 10 A/m^2^ (**G3**) and electric fields of ~ 10–50 V/m (**G7**) would reach the hippocampus. The strong current and electric field at the SVZ (~ 1–10 A/m^2^, ~ 10 V/m) are presumably due to interface of high conductivity, relatively low permittivity of CSF and less conductive, higher permittivity brain parenchyma (**G2**, **G4**, **G6**, **G8**)

To assess the plausibility of using iACS to stimulate the SVZ and hippocampus, we used a finite element method (FEM) to approximately estimate the distribution of currents and electric fields in a three-dimensional mouse brain model (Fig. 1F). Our model is based on a 3D C57BL/6 mouse brain atlas built from MRI and Nissl histology, which consists of 39 different brain segments (Fig. 1F) [43]. We assigned the electrical conductivity and relative permittivity (at 40 Hz, stimulation frequency used in our study) to these segments [44] and rendered the 3D model so it contains a total of 189 × 236 × 152 voxels with voxel resolutions ~ 100 × 100 × 100 μm^3^. We used the Sim4Life platform (Zurich MedTech AG) to perform a quasi-electrostatic FEM simulation to calculate the electric current distribution in the brain model. The simulation calculates the ohmic current, which is suitable for the stimulation frequency used in our study (40 Hz), as the displacement current can be considered negligible.

Modeled electrodes were placed over the dura through a craniotomy hole (Fig. 1E), because electrodes placed on the dura have better control of the electric field/current delivery than electrodes placed over the skin and skull [45, 46]. As the available mouse atlas does not contain cerebral spinal fluid (CSF) layer surrounding the brain and the dura, we added a dura layer of 300 μm (Fig. 1F5) and CSF 43 μm (Fig. 1F6) [42]. Multiple simulations of the iACS current were calculated using electrodes placed at different positions in order to identify electrode positions that would maximally stimulate the SVZ (Fig. 1F9) and hippocampus (Fig. 1F10).

### Electrode placement

The electrode placement was performed on WT and 5xFAD mice 1 day before iACS. The mice were anesthetized with 2% (v/v) isoflurane in oxygen. The mice were then placed on a thermostatically controlled warm pad with a rectal thermometer in a stereotaxic frame. The mice were monitored for depth of anesthesia using a foot pinch and anesthesia administration was increased if necessary. The scalp was shaved and sanitized. Two burr holes with a diameter of 1.5 mm were made on the skull at the following positions relative to bregma: anterior-posterior (AP) = − 2 mm, and medial-lateral (ML) = 4 mm left for one electrode and 4 mm right for the other electrode. Two sterilized stainless steel screws (#0-80, × 1.6 mm) were placed into the burr holes as the electrodes (Fig. 1A–D). The screws only touched the dura but did not contact into the brain tissue (Fig. 1E). After electrode implantation, the electrode screws and scalp incision were fixed and covered with dental cement. For the analgesic regimen, the mice received the subcutaneous carprofen at 2 mg/kg at the time of surgery. The mice were assessed twice daily in the following 2 days post-surgery, and carprofen (2 mg/kg) was administered if mice showed signs of pain or stress.

### Intracranial AC stimulation in 5xFAD mice

The iACS was delivered through the screw electrodes 1 day post-implantation. Prior to conducting iACS, the mice were anesthetized with ketamine/xylazine (90/4.5 mg/kg i.p.). The iACS was performed with the following parameters: 40 Hz with an amplitude of 100 μA (signal produced and monitored by the Neuroelectrics® Starstim®), for 1 h each day on Monday, Wednesday, Friday, and Sunday of the first week; no stimulation in the 2nd week; for 1 h on Monday of the 3rd week; in the 4th week for 1 h on Monday, and then for 1 h on Sunday followed immediately by euthanization and cardiac perfusion with cold 0.1 M PB solution, followed by 4% paraformaldehyde (PFA) for brain tissue collection. The WT and 5xFAD control mice received the same anesthesia, surgery, and screw implantation. During sham control stimulation, no iACS was applied after the anesthesia. During the 1-month iACS treatment period, the body weight and neurological behavior were monitored once each day to guarantee the safety of iACS on 5xFAD and WT mice.

To validate and exclude noise and DC leak, we examined the output wave with the oscilloscope as shown in Fig. S1A. When we set the iACS at 40 Hz with an amplitude of 100 μA for the 5xFAD mice as an example test, the EEG power spectral density analysis also showed an accurate oscillation peak at 40 Hz (Fig. S1B). The waveform and spectral analysis both indicated that the stimulation system we used have produced the accurate and desired frequency, without noise/harmonics.

### Brain tissue fixation and sectioning

The euthanized mice (5 mice for each group) received a cardiac perfusion and fixation with ice-cold 0.1 M PB solution and 4% PFA. The brains were then dissected and incubated in 4% PFA at 4 °C for 3 days before transferring into a 30% (v/v) sucrose solution at 4 °C for another 3 days. After this cryoprotection period, the brains were frozen with dry ice and coronally sectioned at 40-μm intervals with a cryostat microtome. To detect neurogenesis in the SVZ and hippocampus, previously published methods were used [11, 47]. Specifically, coronal brain sections were collected between AP + 1 and − 0.5 mm (thickness 1.5 mm) from bregma (including the SVZ) and between AP − 1.2 and − 2.7 mm (thickness 1.5 mm) from the bregma (including the hippocampus).

### Immunofluorescence

Brain slices were fixed in 4% PFA for 30 min and then permeabilized in 0.1% Triton X-100 (Sigma-Aldrich, MO, USA) for another 30 min, as we have previously described [48]. After blocking non-specific proteins with 3% bovine serum album (BSA) in 0.1 M phosphate-buffered saline (PBS) solution at room temperature for 1 h, the slices were incubated with the primary antibodies: Ki67 (1:1000, 9129S, Cell Signaling Technology), Nestin (1:1000, #4760S, Cell Signaling Technology), DCX (1:1000, #4604S, Cell Signaling Technology), at 4 °C, overnight. After rinsing with PBS, the slices were transferred into 3% BSA-PBS solution containing goat anti-mouse/rabbit (Alex Fluor 594/488, 1:1000, #A-11005, #A-11034, Invitrogen) secondary antibodies. 4′,6-Diamidino-2-phenylindole (DAPI) was applied to label nuclei. For the negative control, the same procedures were performed with the adjacent slices without primary antibody incubation. The fluorescence was detected with confocal laser-scanning microscopy (Leica SP8 STED 3X microscope with × 20X and × 63 1.4 NA objectives).

### Quantification of immunopositive cells

The immunopositive cell counting was performed with ImageJ software, for analysis of neurogenesis in the SVZ and hippocampus. The Ki67+, Nestin+, and DCX+ cells were respectively determined at 6 section intervals (240 μm apart) using a × 20 objective. Within each section, we picked 3 views of area 200 × 200 μm^2^ for cell counts. The cell numbers in the SVZ and hippocampal regions of each animal were calculated and averaged to obtain the group mean and standard deviation.

### Statistics

Data analysis was performed using SPSS software (version 26; SPSS, Chicago, IL), which adheres to a general linear model. The alpha level for type I error was set at 0.05 for rejecting null hypotheses. Data were expressed as mean ± standard error of the mean (SEM). Neurogenesis cell counts from Ki-67, Nestin, and DCX staining were separately analyzed by one-way ANOVA for each group, followed by a Tukey’s honestly significant difference post hoc analysis for the WT, 5xFAD, and 5xFAD+iACS group comparation.

## Results

### Modeled iACS in the SVZ and hippocampus

We first estimated how likely iACS would be able to deliver electric stimulation to the SVZ and hippocampus in mice. The distribution of current density and electric field in a 3D mouse brain model (Fig. 1F) was approximately simulated using a finite element method to optimize the positions of the electrodes on the dura surface (Fig. 1). The screw electrodes were implanted through a cranial hole, which were in contact with the dura while keeping the dura and brain tissue intact (Fig. 1B–E). The simulation showed a strong current density and electrical field in the SVZ and hippocampus when the electrodes were placed at the dura surface with coordinates at AP: − 2 mm and ML: one 4 mm left and one 4 mm right to bregma (Fig. 1A–C). As the current originates from the electrodes, the strongest electric field occurs within the cortical regions closest to the electrodes, yielding a maximum electric field of ~ 100 V/m (Fig. 1G5–G8). The electric field magnitude gradually decreases deeper into the brain (Fig. 1G5–G8). Importantly, the maximum current density (~ 10 A/m^2^, Fig. 1G1–G4) is lower than what would be required to induce lesions, which has been reported above 20 A/m^2^ [49–51]. Compared to most other subcortical regions, the hippocampus is close to the electrodes, thus receiving a relatively strong electric field (~ 10–50 V/m) (Fig. 1F7). Although the SVZ is deep in the brain, it too experiences a strong electric field (~ 10 V/m), presumably due to the low permittivity of the CSF in the ventricles compared to the surrounding parenchyma. Furthermore, there appears a strong cluster of current in the SVZ, presumably due to the high electrical conductivity of CSF in SVZ than the surrounding parenchyma (Fig. 1G2, G4).

In the process of optimizing the position of the electrode pair so the electrical stimulation can be delivered to the hippocampus and SVZ most effectively, we chose a few other candidate coordinates (I–IV, supplementary Fig. S2) on the dura surface for the electrode pair and simulated the distribution of the electrical field and current density. These candidate coordinates were chosen because they are in proximal distance to the hippocampus and SVZ. In all these electrode coordinates, the electrical field and current could be delivered through the dura and cortex and gradually decreased deeper into the brain. However, the electric field distributions with positions I–IV were more focused to the electrodes and therefore cover less regions in deeper brain, including the hippocampus and SVZ, presumably due to a shorter distance between the two electrodes. Comparing with I–IV, the optimized electrode position (Fig. 1, X and Y in supplementary Fig. S2) yielded better coverage to the deep brain, and the desirable electric field strengths at both target regions of the hippocampus and SVZ (Supplementary Table S1 and S2). Based on the simulation result, we placed the electrodes at the above-noted coordinates, which would maximally target the electrical stimulation to the SVZ and hippocampus.

### Defective neurogenesis in the SVZ and hippocampus of the 5xFAD mouse

To characterize neurogenesis in the SVZ and hippocampus of the 5xFAD control mice (who received sham stimulation), we compared the neurogenesis in WT and 5xFAD control mouse brains using Ki67 (cell proliferation marker), Nestin (neural stem cell/neural precursor cells marker), and DCX (neuronal precursor marker). One-way ANOVAs revealed a significant difference between the following treatment groups: Ki67 in the SVZ and hippocampus [*F*(2,12) = 57.3; *P* < 0.001] and [*F*(2,12) = 13.1; *P* = 0.001], respectively; Nestin in the SVZ and hippocampus [*F*(2,12) = 17.7; *P* < 0.001] and [*F*(2,12) = 93.0; P < 0.001], respectively; and DCX in the SVZ and hippocampus [*F*(2,12) = 78.2; *P* < 0.001] and [*F*(2,12) = 26.4; *P* < 0.001], respectively.

**Fig. 2:**
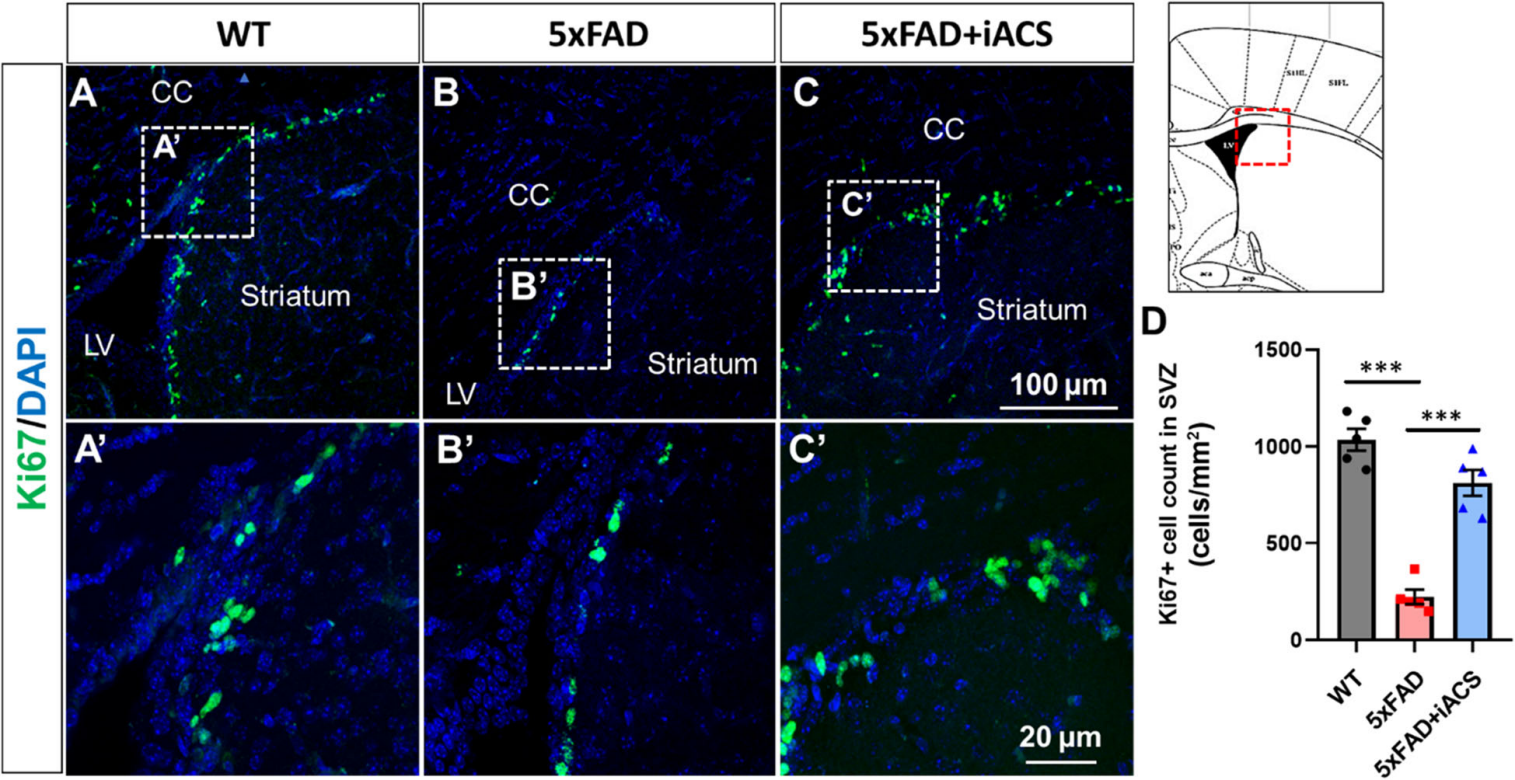
iACS increased the neural proliferation in the SVZ of 5xFAD mouse brain. **A**–**C** Cells positive for proliferation marker Ki67 were reduced in the SVZ of 5xFAD mouse brains. iACS increased Ki67-positive cells. Scale bar 100 μm (**A**–**C**) and 20 μm (**A’**–**C’**). **D** The numbers of cells positive for Ki67 were significantly reduced in the SVZ of 5xFAD mouse brain and were significantly recovered following iACS. ***P < 0.001. *n* = 5 mice for each group

**Fig. 3:**
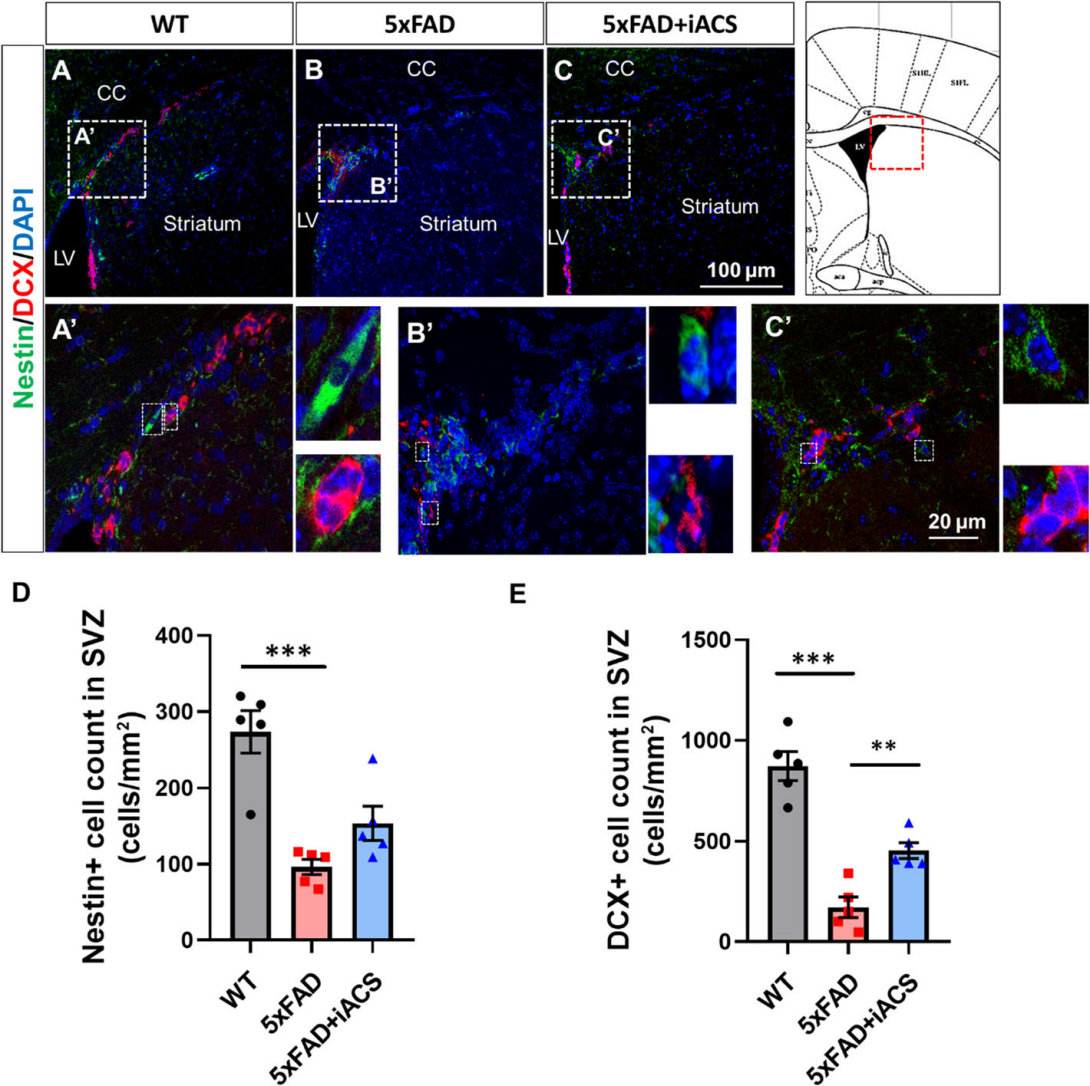
iACS increased the neurogenesis in the SVZ of 5xFAD mouse brain. **A**–**C** Cells positive for Nestin and DCX were scarcer in the SVZ of 5xFAD mouse brains than that of WT control brain. iACS increased both cells positive for Nestin or DCX in the SVZ of 5xFAD mouse brains. Scale bar 100 μm (**A**–**C**) and 20 μm (**A’–C’**). **D, E** The numbers of cells positive for neural precursor cell marker (Nestin, **D**) or neurogenesis marker (DCX, **E**) were significantly reduced in the SVZ of 5xFAD mouse brain and were recovered following iACS. **P < 0.01, ***P < 0.001. *n* = 5 mice for each group

The 5xFAD control brains had significantly fewer Ki67-positive cells (Fig. 2B, B’) than that in the age-matched WT brain (Fig. 2A, A’). Specifically, the average number of Ki67-positive cells in the SVZ of 5xFAD control mice was 224 ± 37, and thus significantly lower than the WT control (1036 ± 57, *P* < 0.001) (Fig. 2D). Cells positive for Nestin and DCX were also decreased in the SVZ of 5xFAD control mice (Fig. 3B, B’), compared to the age-matched WT control (Fig. 3A, A’). Specifically, in the SVZ of aged-matched WT control brain, the average numbers of Nestin- and DCX-positive cells were 273 ± 28 and 873 ± 71, respectively. In the SVZ of the 5xFAD control mice, the numbers were significantly decreased to 96 ± 10 (Nestin+, *P* < 0.001) and 172 ± 51 (DCX+, *P* < 0.001) (Fig. 3D, E).

**Fig. 4:**
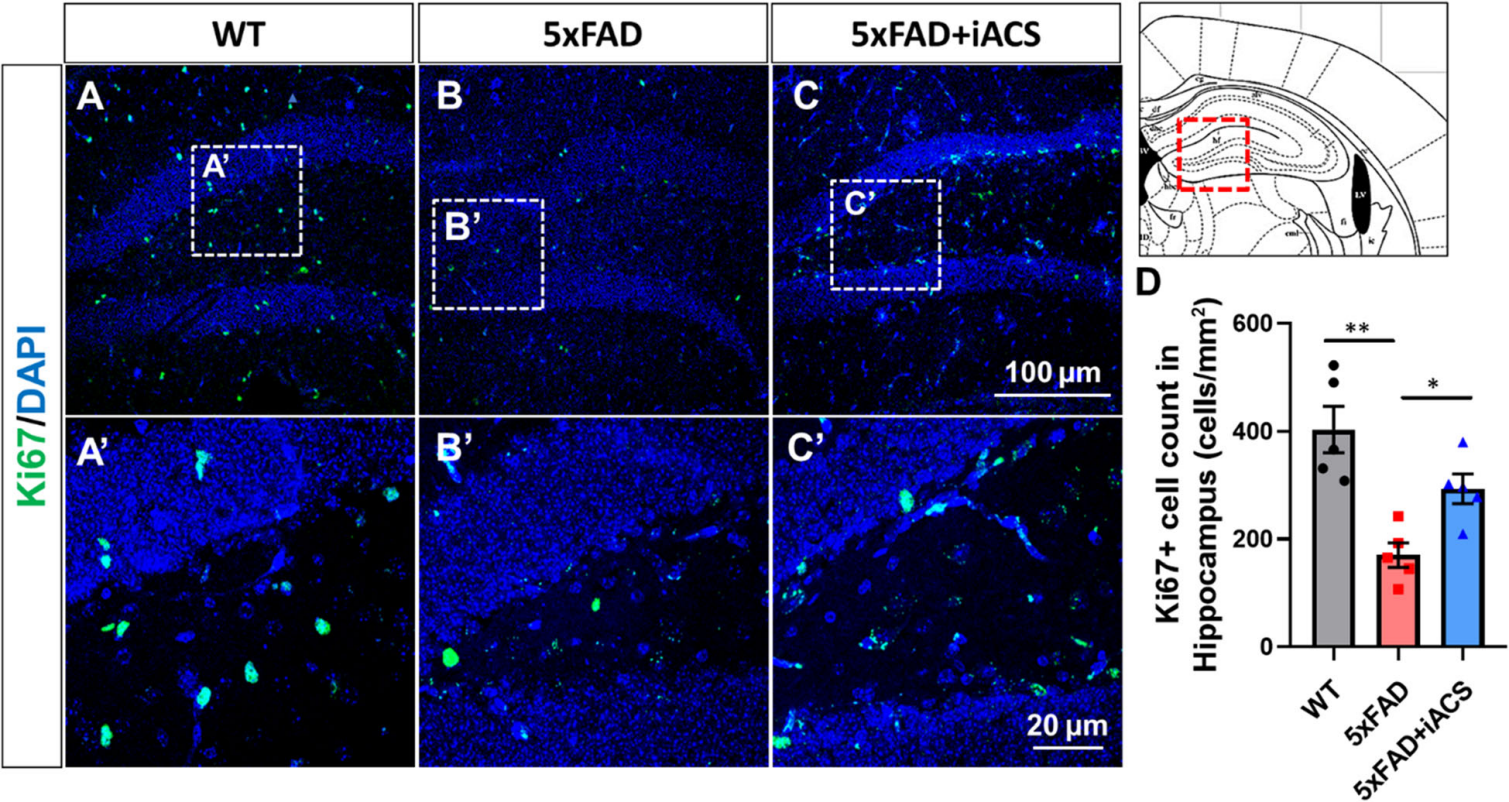
iACS increased the neural proliferation in the hippocampus of 5xFAD mouse brain. **A**–**C** Cells positive for proliferation marker Ki67 were reduced in the hippocampus of 5xFAD mouse brains. iACS increased Ki67-positive cells. Scale bar 100 μm (**A**–**C**) and 20 μm (**A’**–**C’**). **D** The numbers of cells positive for Ki67 were significantly reduced in the hippocampus of 5xFAD mouse brain and were significantly recovered following iACS. *P < 0.05 and **P < 0.01. *n* = 5 mice for each group

**Fig. 5:**
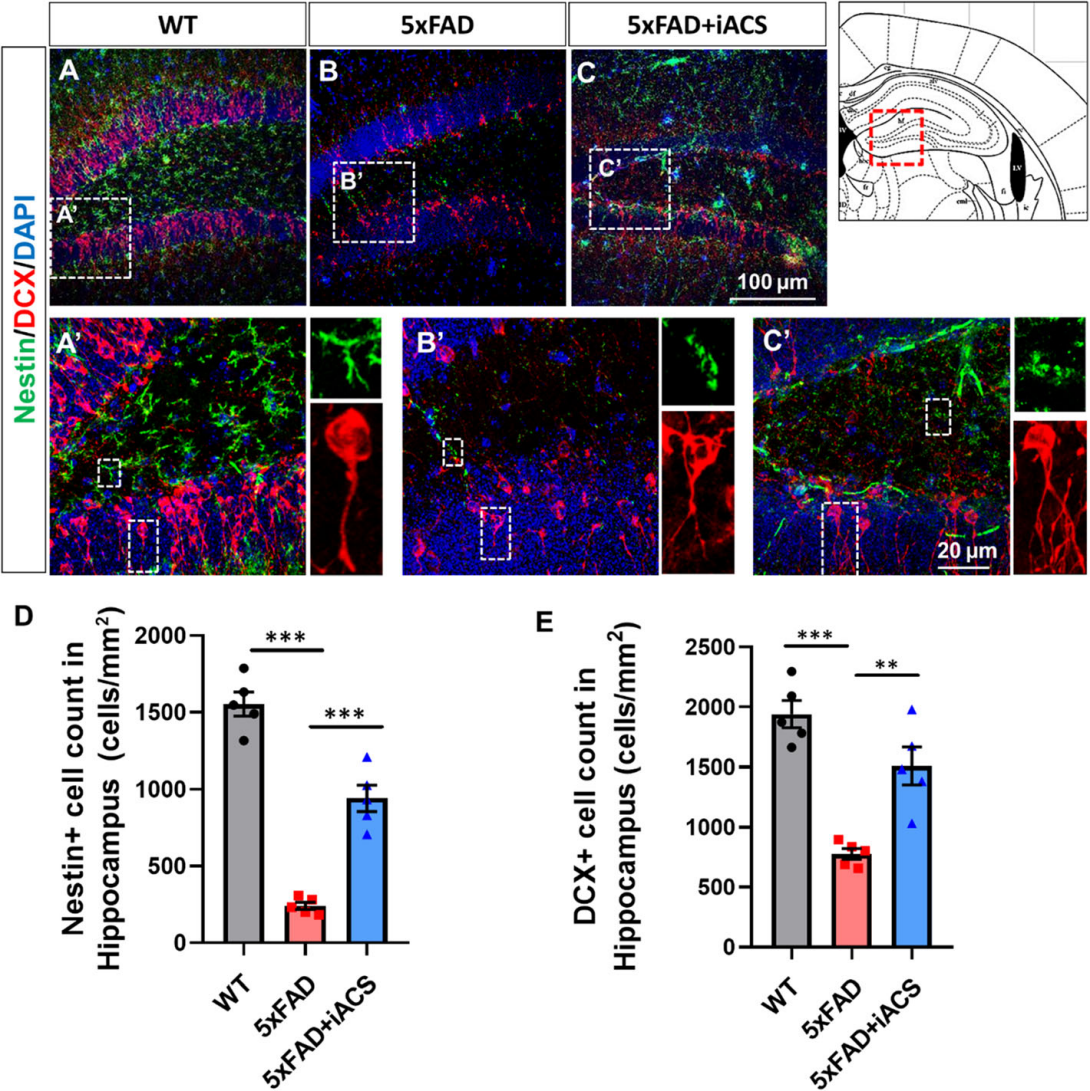
iACS increased the neurogenesis in the hippocampus of 5xFAD mouse brain. **A**–**C** Cells positive for Nestin and DCX were scarcer in the hippocampus of 5xFAD mouse brains than that of WT control brain. iACS increased both cells positive for Nestin or DCX in the hippocampus of 5xFAD mouse brains. Scale bar 100 μm (**A**–**C**) and 20 μm (**A’**–**C’**). **D**, **E** The numbers of cells positive for neural precursor cell marker (Nestin, **D**) or neurogenesis marker (DCX, **E**) were significantly reduced in the hippocampus of 5xFAD mouse brain and were significantly recovered following iACS. **P < 0.01 and ***P < 0.001. *n* = 5 mice for each group

We also determined levels of deficient neurogenesis in the dentate gyrus (DG) of the 5xFAD hippocampus (Fig. 4B, B’) compared to the age-matched WT brain (Fig. 4A, A’). The number of Ki67-positive cells in the hippocampus from 5xFAD control mice was 170 ± 23, significantly lower than that in the hippocampus from WT mice (403 ± 43, *P* = 0.001) (Fig. 4D). Nestin- and DCX-positive cells were also significantly less in the hippocampus of 5xFAD control mice (Fig. 5B, B’) than that from aged-matched WT mice (Fig. 5A, A’). There were 241 ± 24 Nestin+ cells in the hippocampus of 5xFAD control mice, significantly lower than that from WT control (1554 ± 78, *P* < 0.001) (Fig. 5D). The number of DCX+ cells was 777 ± 45 in the hippocampus from 5xFAD control mice, a marked drop from 1941 ± 113 in the hippocampus of WT mice (*P* < 0.001) (Fig. 5E).

### iACS promoted neurogenesis in the SVZ and hippocampus in the 5xFAD mouse

To determine the effects of iACS on neurogenesis, 5xFAD mice who received iACS were compared to both WT mice and 5xFAD control mice who received sham stimulation. In the SVZ, iACS significantly increased the number of Ki67+ cells in 5xFAD brain (Fig. 2C, C’) vs. the 5xFAD control (Fig. 2B, B’). The number of Ki67-positive cells in the SVZ of 5xFAD mice following iACS (812 ± 67, *P* < 0.001) was significantly higher than that of 5xFAD control, and almost approaches the levels as in the aged-matched WT brain (Fig. 2D). iACS also increased Nestin- and DCX-positive cells significantly in the SVZ of 5xFAD mice (Fig. 3C, C’) compared to the 5xFAD control brain (Fig. 3B, B’). Following iACS, counts of Nestin-positive cells (154 ± 22, *P* = 0.148) and counts of DCX-positive cells (454 ± 39, *P* = 0.009) were about twice and five times, respectively, of that in the SVZ of 5xFAD control mice (Fig. 3D, E).

iACS also significantly increased cells positive for Ki67 in the DG (Fig. 4C, C’) compared to 5xFAD control mice (Fig. 4B, B’). Counts of Ki67-positive cells in the hippocampus of 5xFAD mice after iACS (293 ± 27, *P* = 0.048, 5xFAD + iACS vs. 5xFAD control) recovered to almost the same level found in the DG of the age-matched WT brain (Fig. 4D). Nestin- and DCX-positive cells were also significantly increased in the hippocampus of 5xFAD mice following iACS (Fig. 5C, C’), compared to that in the brain of 5xFAD control mice (Fig. 5B, B’). The counts of Nestin-positive cells (942 ± 85, *P* < 0.001) and counts of DCX-positive cells (1510 ± 157, *P* = 0.002) were significantly increased from that in the hippocampus of 5xFAD control mice (Fig. 5D, E).

## Discussion

In this report, we demonstrated that iACS delivered through electrodes positioned on the surface of the brain and over the dura can effectively stimulate the SVZ and hippocampus in mice. Importantly, this stimulation of the SVZ and hippocampus increases neurogenesis in these regions within a mouse model of Alzheimer’s disease. These findings suggest a potential intracranial stimulation approach to boost neurogenesis in brains progressing towards Alzheimer’s disease.

Alzheimer’s disease (AD) is the most prevalent cause of dementia, which is among the leading causes of severe and long-term disability worldwide. Unfortunately, treatment options for AD are very limited and are ineffective long term. Decreased neurogenesis has been reported in both the human Alzheimer’s brain and animal models of AD [4, 52]. In an AD mouse model (5xFAD), we observed defective neurogenesis in the hippocampus in an earlier age (4-month-olds) (Figs. 4 and 5), consistent with published literature describing that neurogenesis is defective in much older 5xFAD mice (7- and 12-month-olds) [52, 53]. Although studies of neurogenesis in the SVZ from Alzheimer’s patients and experimental animals are less reported [52], our results suggest that neurogenesis is also defective in the SVZ (Figs. 2 and 3). Importantly, our results demonstrated that in 5xFAD mice, defective neurogenesis happens earlier and becomes significant in 4-month-old animals. Interestingly, recent research in human AD brains has corroborated animal models in showing that humans with AD also exhibit deficient neurogenesis [4]. As such, defective neurogenesis is suggested to play an important role in the progress of AD [54].

To remediate declines in neurogenesis, we assessed iACS in a mouse model of AD. Based on a computational model, iACS electrodes were placed to maximally stimulate the SVZ and hippocampus. iACS was then applied in several 1-h sessions over the course of a month. The body weights and neurological performances (active, alert, limb draw withdrawal from a pinch) were both monitored to confirm that the mice were kept in healthy and active state pre- and post-surgery and iACS treatments, following the animal care standard procedures. Our records showed no body weight loss or poor neurological performance during the whole experiment period, indicating a safe treatment with the iACS. Compared to 5xFAD mice that received sham (control) stimulation, iACS increased all three of our markers of neurogenesis (Ki67, Nestin, DSX) within the hippocampus and SVZ. These results provide important preliminary evidence for the potential of iACS to serve as a therapeutic treatment for AD.

Despite these promising results, much additional research is necessary to develop a pathway for the use of iACS as a therapeutic modality. Much of this research falls into one of five categories: (1) safety, (2) mechanisms of action, (3) behavioral effects, (4) sustainability, and (5) translation to humans. In terms of safety and mechanisms of action, potential mechanisms of deep brain stimulation intending to induce neurogenesis include injury to brain tissue, inflammation, cytokine, and growth factor release [55]. The iACS parameters used in our experiment did not elicit histological evidence of neuronal damage (Fig. S3) and, therefore, prove to be safer than deep brain stimulation and would exclude injury effects as a mechanism of action. Future research will also focus on whether facilitating neurogenesis improves functional outcomes, such as enhanced memory. Indeed, deep brain electrical stimulation facilitates both neurogenesis and improves memory [34]. Thus, iACS-induced neurogenesis will likely show similar benefits on behavior. Another open question is whether these effects will be sustained. Here, we tested 3-month-old 5xFAD mice. However, neurogenesis continues to deteriorate and becomes most severe in 5xFAD mice between 7 and 12 months of age [52, 53]. It will be important to understand how long iACS treatments will be necessary to sustain neurogenesis and delay (or reverse) the progression to AD. Finally, feasibility studies are necessary to translate this research to humans. Based on our numerical simulation, the maximum modeled current density (~ 10 A/m^2^) and electric field magnitude (~ 100 V/m) are approximately 100 times higher than the typical current density (~ 0.1 A/m^2^) and electric field magnitude (~ 0.4 V/m) applied in human transcranial electrical stimulation. Yet, the stimulation applied here is still much lower than the intensity applied during human electroconvulsive therapy (ECT) [56]. Future research should assess whether comparable effects in mice may be obtained with lower intensities that are more comparable to typical iACS applications in humans, which are known to be highly tolerable and safe.

Another hurdle in the translation of this technique to humans is that human iACS is applied at the surface of the scalp. Unfortunately, most electric currents shunt outside the brain when electrodes are placed on the scalp and skull surface [45]. Even with electrodes placed in contact with the dura, most of the current distributes in the cerebral spinal fluid layer (Fig. 1G). Yet, electric currents of significant strength may reach the hippocampus and cluster at the SVZ in mice (Fig. 1G3, G4). Prior simulations and measurements have confirmed that sizable electric fields could reach 3–4 mm from an electrode on the dura [45], which overlaps with the hippocampus. Our own simulations are consistent with this observation. More importantly, our FEM estimation shows that currents and electric fields cluster at the SVZ (Fig. 1G2, G4, G6, G8), because the SVZ is located between the cerebral spinal fluid in the ventricles with a high electrical conductivity and a low permittivity, and the brain parenchyma with a low electrical conductivity and a high permittivity. This anatomical feature suggests a physical basis for the ability of iACS to stimulate the SVZ, which is located deeper in the brain. Furthermore, even in larger brains, such as human, similar electrical clustering effects in SVZ are expected to happen. These clustering effects can then be exploited with improved simulation techniques by considering anisotropic conductivity, and further consideration of detailed individual anatomy. It should be noted that our estimation is based on a generalized 3D mouse brain model (Fig. 1F), which is an oversimplified approximation at this stage, for example, some very obvious structures, such as blood vessels, orientation of white matters, and glymphatic system were not considered. Their results therefore may be different from the exact mouse brain. Although our modeling approach takes into account a more detailed structure of the brain than previous reports and provides a good approximation of the iACS current and electric field distribution (Fig. 1), the model could be improved with individual mouse MRIs, including the anisotropy of conductivity of different tissues, and validating results with direct experimental measurements of the electrical field distribution inside the brain. These are all important topics to be studied in the future. For more non-invasive stimulation in humans, the scalp, skull, vasculature, and anisotropic features of the head and brain tissues should be included in an individualized model using MRI data. This will help account for heterogeneity of the brain structure, which can be compromised by diseases and injuries [57–59]. Nevertheless, the simulation approach presented here could serve as an initial step to facilitate placement of electrodes on the patient head and in experimental animals, based on the estimated strength of the resulting electric field in the targeted brain regions. Moreover, improving the model will help to provide more precise mapping of the current and field distribution, which may in turn be used to guide electrode placement and provide information regarding how heterogeneity in the brain affects the induced electric fields and subsequent stimulation effects.

One interesting point we observed was the variable degree of neurogenesis-enhancing effects in the hippocampus compared to the SVZ. The iACS increased cell proliferation in SVZ by 3.6-fold over the sham stimulation control 5xFAD mice (Fig. 2D), but barely increased 1.7-fold in the hippocampus (Fig. 4D). This difference is consistent with the effect of iACS on the number of newborn neurons (DCX+), which increased 2.6-fold in the SVZ, compared to a 1.9-fold increase in the hippocampus (Fig. 3E vs. Fig. 5E, respectively). The pronounced effect in the SVZ may be due to larger clustering of currents at the SVZ as suggested by our simulation (Fig. 1G2, G4), although we cannot discount possible biological differences in these regions. Additional research is required to assess the effects of stimulation intensity on neurogenesis.

## Conclusions

**Fig. 6:**
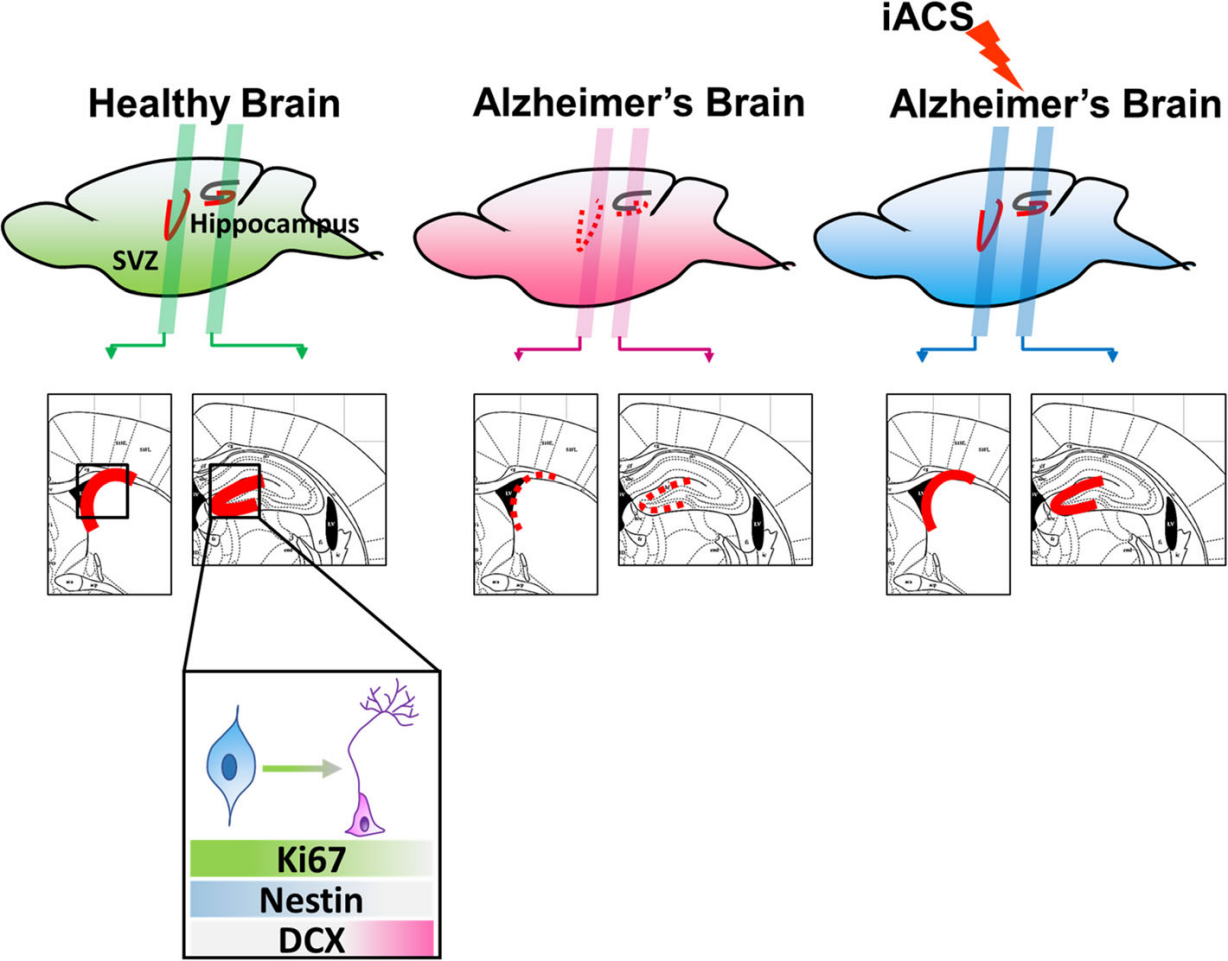
Summary diagram to show iACS enhanced neurogenesis in the hippocampus and SVZ of Alzheimer’s brain

Here, we report intracranial electrical stimulation that effectively boosted neurogenesis in the hippocampus and SVZ in 5xFAD mice, an AD model (Fig. 6). Our results suggest that iACS is a promising, minimally invasive technique to stimulate the hippocampus and SVZ in the mouse brain. Appropriate iACS can significantly facilitate neurogenesis in the brain and offer a potential new approach for the treatment of AD.

## Supplementary information

**Additional file 1: Fig. S1.** Output waveform and spectrogram of the 40 Hz stimulation. (**A**). Output waveform of the 40 Hz iACS, by oscilloscope. (**B**). EEG power spectral densities of 5xFAD mouse brain, pre-, inter- and post-iACS. **Fig. S2.** Computer simulation was used to estimate the current densities (1–4, A/m^2^) and electric field strengths (5–8, V/m) in different brain regions, with different electrode positions. **Fig. S3.** iACS did no damage to the neurons and brain of 5xFAD mouse.

**Additional file 2: Table S1.** Average current densities at the hippocampus and SVZ with different electrode positions from Fig. S1. **Table S2.** Average electric fields at the hippocampus and SVZ with different electrode positions from Fig. S1.

## Data Availability

The datasets used and/or analyzed during the current study are available from the corresponding authors on reasonable request.

## Abbreviations

AD: Alzheimer’s disease
5xFAD: The five-familial Alzheimer’s disease transgenic mouse model
DCX: Doublecortin
SVZ: Subventricular zone
iACS: Intracranial alternating current stimulation
WT: Wild type
NPCs: Neural precursor cells
FEM: Finite element method
CSF: Cerebral spinal fluid
AP: Anterior-posterior
ML: Medial-lateral
PFA: Paraformaldehyde
PBS: Phosphate-buffered saline
DAPI: 4′,6-Diamidino-2-phenylindole
