# Supplementary material for: Intracranial alternating current stimulation facilitates neurogenesis in a mouse model of Alzheimer’s disease
Source: Alzheimers Res Ther. 2020 Jul 23;12:89. doi: 10.1186/s13195-020-00656-9 (PMC7376967; doi:10.1186/s13195-020-00656-9)
Supplement: Supplementary file 1 — Additional file 1: Fig. S1. Output waveform and spectrogram of the 40 Hz stimulation. (A). Output waveform of the 40 Hz iACS, by oscilloscope. (B). EEG power spectral densities of 5xFAD mouse brain, pre-, inter- and post-iACS. Fig. S2. Computer simulation was used to estimate the current densities (1–4, A/m2) and electric field strengths (5–8, V/m) in different brain regions, with different electrode positions. Fig. S3. iACS did no damage to the neurons and brain of 5xFAD mouse. [file 13195_2020_656_MOESM1_ESM.docx]

**Supplementary Figure S1**

**
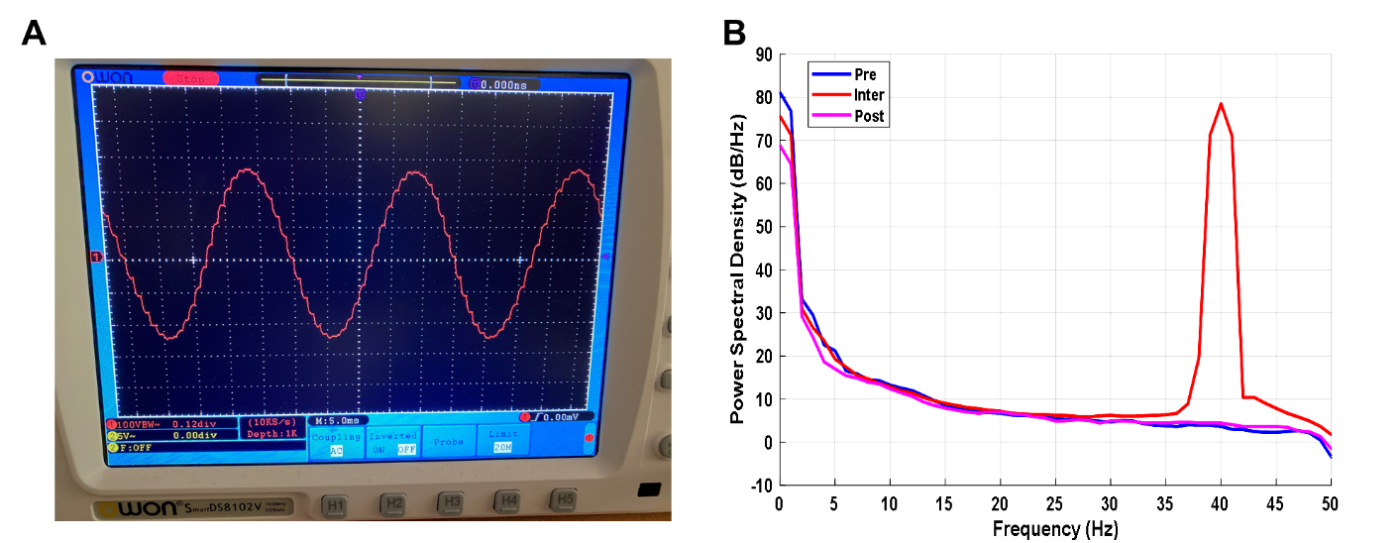
**

**Fig. S1.** Output waveform and spectrogram of the 40 Hz stimulation. **(a)**. Output waveform of the 40 Hz iACS, by oscilloscope. **(b)**. EEG power spectral densities of 5xFAD mouse brain, pre-, inter- and post-iACS.

**Supplementary Figure S2**


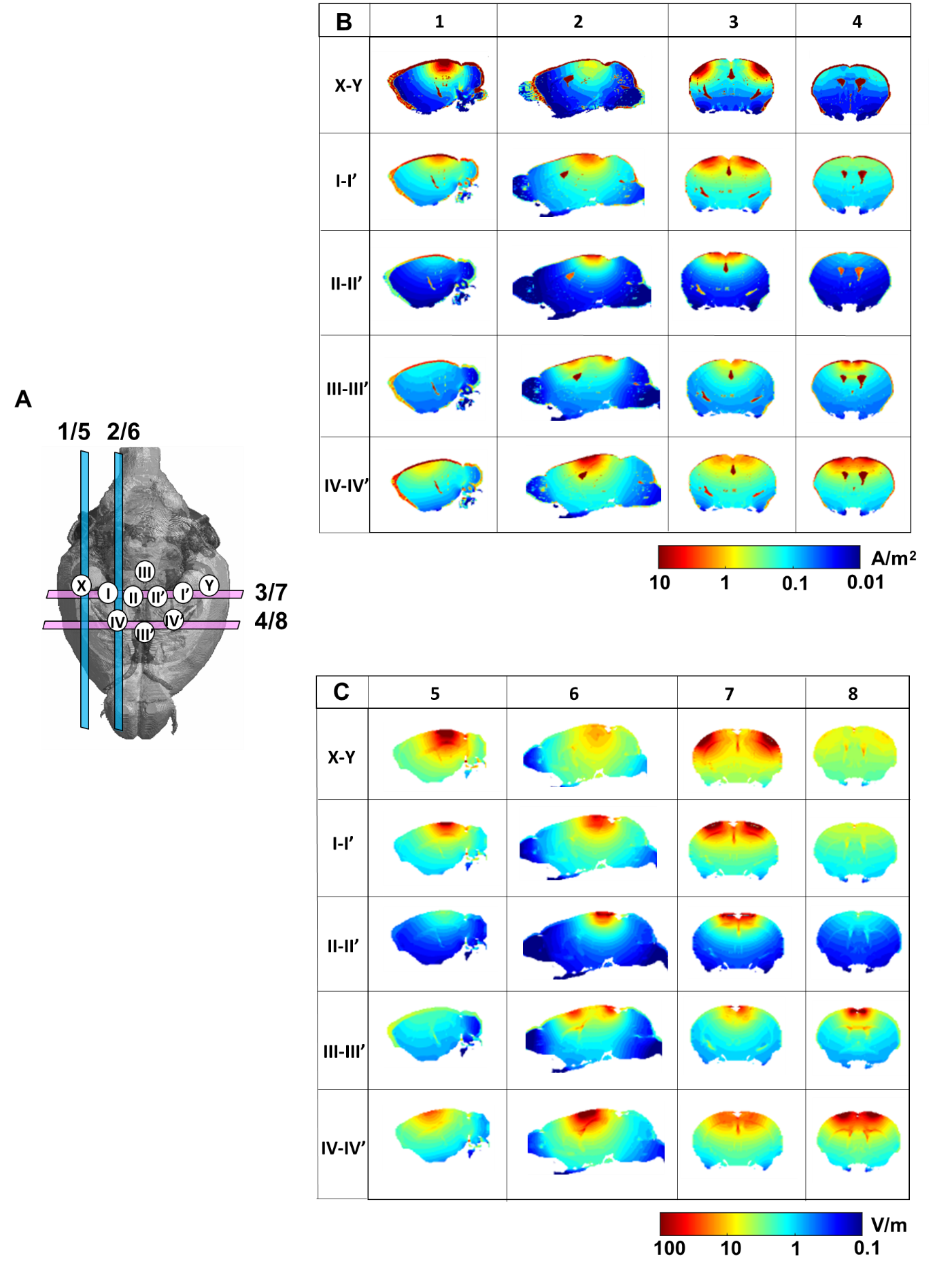


**Fig. S2.** Computer simulation was used to estimate the current densities (**1-4**, A/m^2^) and electric field strengths (**5-8**, V/m) in different brain regions, with different electrode positions. (**a**). Electrodes were placed at positions: I-IV for output current and electric field optimization. I-I’: AP = -2 mm, ML = 2.5 mm (left and right); II-II’: AP = -2 mm, ML = 1 mm (left and right); III: AP = -3.8 mm, ML = 0 mm; III’: AP = 1 mm, ML = 0 mm; IV-IV’: AP = 0.5mm, ML = 1.5 mm (left and right). X and Y were the electrode positions in **Fig. 1g**. (**b**). The current density distributions with different electrode positions. (**c**). The electric field distributions with different electrode positions.

**Supplementary Figure S3**


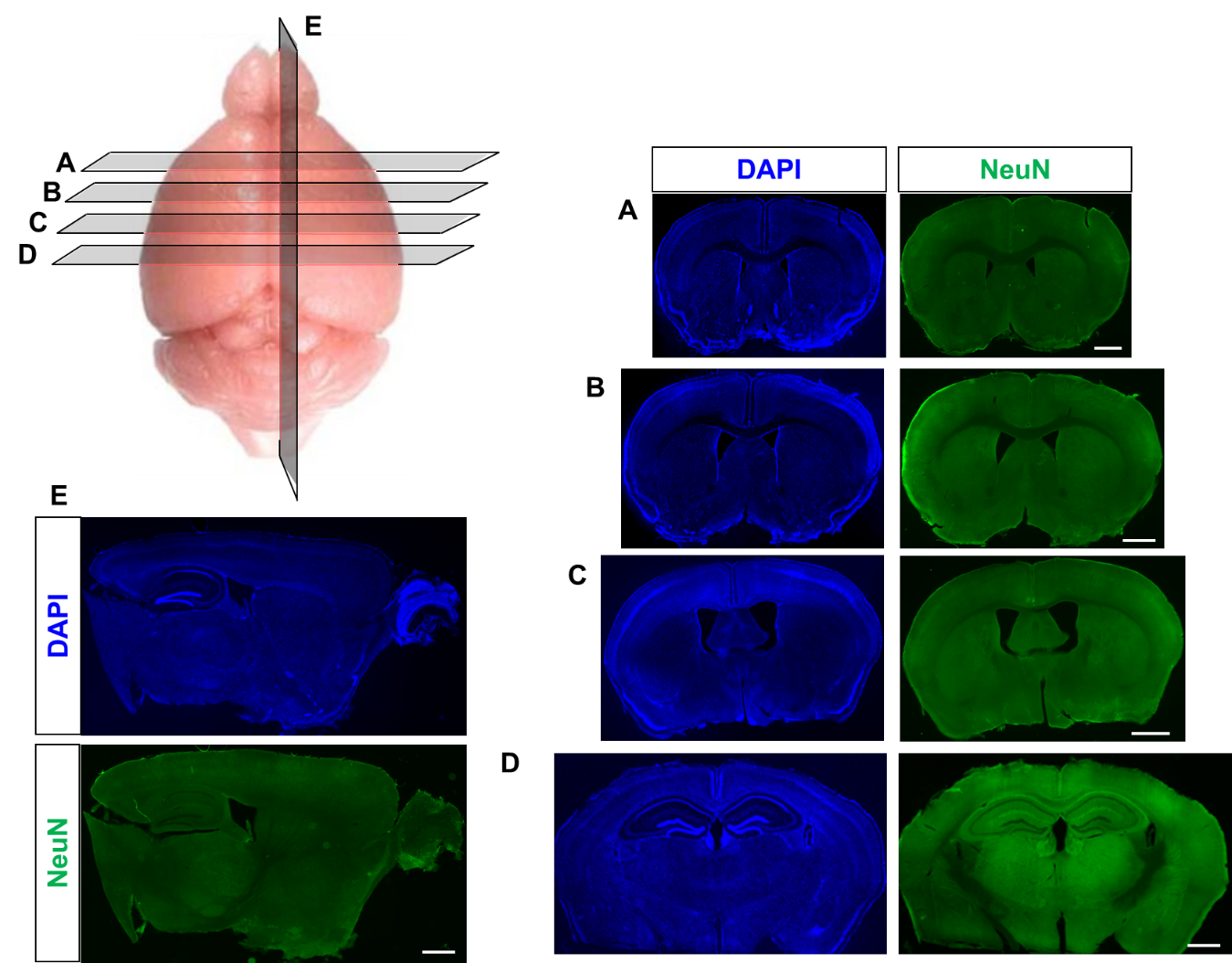


**Fig. S3.** iACS did no damage to the neurons and brain of 5xFAD mouse. (**a-d**). Coronal slices of the 5xFAD brain with the iACS treatment. (**e**). Sagittal slice of the 5xFAD brain with the iACS treatment. Immunofluorescence of NeuN and DAPI showed no neuronal damage or tissue loss after the one-month iACS treatment on 5xFAD mouse. Scale bars: 1 mm.
