# Supplementary material for: Intracranial alternating current stimulation facilitates neurogenesis in a mouse model of Alzheimer’s disease
Source: Alzheimers Res Ther. 2020 Jul 23;12:89. doi: 10.1186/s13195-020-00656-9 (PMC7376967; doi:10.1186/s13195-020-00656-9)
Supplement: Supplementary file 2 — Additional file 2: Table S1. Average current densities at the hippocampus and SVZ with different electrode positions from Fig. S1. Table S2. Average electric fields at the hippocampus and SVZ with different electrode positions from Fig. S1. [file 13195_2020_656_MOESM2_ESM.docx]

**Supplementary Table S1:** Average current densities at the hippocampus and SVZ with different electrode positions from Fig. S1.

| **Electrodes** | **Coordinate**  **(mm to bregma)** | **Hippocampus (A/m^2^)** | **SVZ**  **(A/m^2^)** |
| --- | --- | --- | --- |
| **X-Y** | AP = -3, ML= 4 mm (Left and right) | 0.9867 | 10.45 |
| **I-I'** | AP = -2, ML = 2.5 (Left and Right) | 0.5124 | 6.085 |
| **II-II'** | AP = -2, ML = 1 (left and right) | 0.0985 | 1.344 |
| **III-III'** | AP = -3.8, ML = 0; AP = 1, ML = 0 | 0.2071 | 8.992 |
| **IV-IV'** | AP = 0.5, ML = 1.5 (Left and Right) | 0.6253 | 19.3616 |

**Supplementary Table S2:** Average electric fields at the hippocampus and SVZ with different electrode positions from Fig. S1.

| **Electrodes** | **Coordinate**  **(mm to bregma)** | **Hippocampus (V/m)** | **SVZ**  **(V/m)** |
| --- | --- | --- | --- |
| **X-Y** | AP = -3, ML= 4 mm (Left and right) | 13.06 | 8.252 |
| **I-I'** | AP = -2, ML = 2.5 (Left and Right) | 6.951 | 4.743 |
| **II-II'** | AP = -2, ML = 1 (left and right) | 1.34 | 1.03 |
| **III-III'** | AP = -3.8, ML = 0; AP = 1, ML = 0 | 2.345 | 6.256 |
| **IV-IV'** | AP = 0.5, ML = 1.5 (Left and Right) | 7.76 | 14.33 |
